# Supplementary figures and images for: Using Participatory Narrative Inquiry to Assess Experiences and Self-Experimentation with Diet Interventions in Inflammatory Bowel Disease Patients
Source: Nutrients. 2024 Nov 24;16(23):4027. doi: 10.3390/nu16234027 (PMC11643754; doi:10.3390/nu16234027)

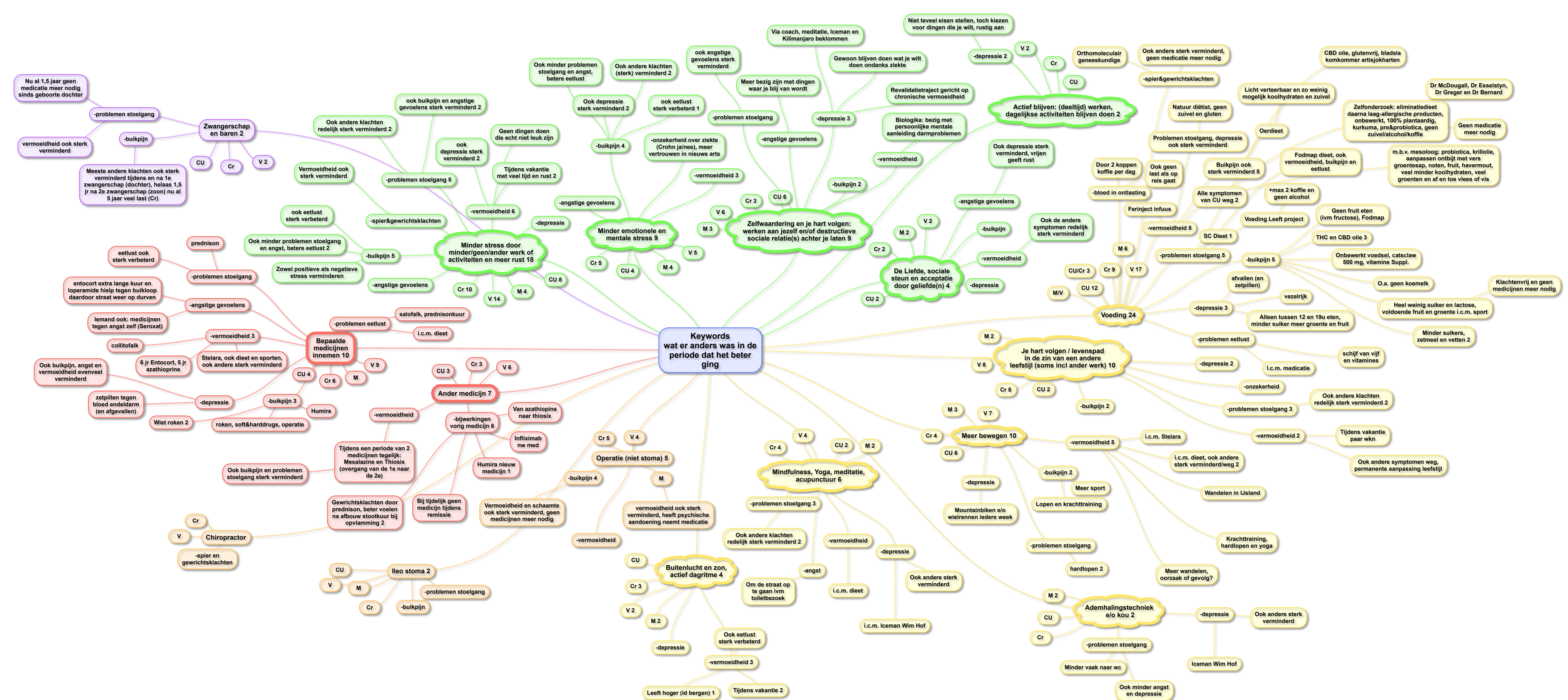

Supplement: Supplementary file 1 [file nutrients-16-04027-s001.zip › nutrients-3313095-Figure S1.pdf]
